# Acquisition of Viewpoint Transformation and Action Mappings via Sequence to Sequence Imitative Learning by Deep Neural Networks

**DOI:** 10.3389/fnbot.2018.00046

**Published:** 2018-07-24

**Authors:** Ryoichi Nakajo, Shingo Murata, Hiroaki Arie, Tetsuya Ogata

**Affiliations:** ^1^Department of Intermedia Art and Science, Waseda University, Tokyo, Japan; ^2^Department of Modern Mechanical Engineering, Waseda University, Tokyo, Japan

**Keywords:** imitative learning, human-robot interaction, recurrent neural networks, deep neural networks, sequence to sequence learning

## Abstract

We propose an imitative learning model that allows a robot to acquire positional relations between the demonstrator and the robot, and to transform observed actions into robotic actions. Providing robots with imitative capabilities allows us to teach novel actions to them without resorting to trial-and-error approaches. Existing methods for imitative robotic learning require mathematical formulations or conversion modules to translate positional relations between demonstrators and robots. The proposed model uses two neural networks, a convolutional autoencoder (CAE) and a multiple timescale recurrent neural network (MTRNN). The CAE is trained to extract visual features from raw images captured by a camera. The MTRNN is trained to integrate sensory-motor information and to predict next states. We implement this model on a robot and conducted sequence to sequence learning that allows the robot to transform demonstrator actions into robot actions. Through training of the proposed model, representations of actions, manipulated objects, and positional relations are formed in the hierarchical structure of the MTRNN. After training, we confirm capability for generating unlearned imitative patterns.

## 1. Introduction

Today there is increased interest in robots capable of working in human living environments. Robot motions are generally preprogrammed by engineers, but it is crucial for robots to learn new actions in work environment contexts if they are to work with humans. One way for robots to learn new actions is imitation, which is the behavioral capability to generate the equivalent actions after the observation of the demonstrator's actions. Imitation is a powerful learning method that humans apply to acquire new actions without resorting to trial-and-error attempts. Hence, robot acquisition of imitative abilities will realize *programming by demonstration* (PbD) (Billard et al., 2008), in which new action skills are acquired from demonstrators without any prior design.

Early studies of imitation learning are related to computational neuroscience, focusing on task-level imitation such as assembly (Kuniyoshi et al., 1994), kendama manipulation (Miyamoto et al., 1996), and tennis serves (Miyamoto and Kawato, 1998). To date, the main approaches to imitative learning have been probabilistic models, reinforcement learning, and neural networks. Among probabilistic models, hidden Markov models realize behavior recognition, generation through imitative learning (Inamura et al., 2004), and imitation of object manipulation (Sugiura et al., 2010). Gaussian mixture models allow robots to imitate human gestures (Calinon et al., 2010). Reinforcement learning has been used for robot acquisition of motor primitives (Kober and Peters, 2010) and applied to task-level learning (Schaal, 1997). By combining reinforcement learning with a Gaussian mixture model, Guenter et al. (2007) achieved robot imitation of reaching movements. Neural network approaches mainly use recurrent neural networks that allow robots to imitate human gesture patterns (Ito and Tani, 2004) and object manipulations (Ogata et al., 2009; Arie et al., 2012).

As another perspective, cognitive developmental robotics (Asada et al., 2009; Cangelosi et al., 2010) has tried to understand the development of the human cognitive abilities through robot experiments based on constructive approaches. In studies focusing on imitative learning, robots were trained to learn imitative tasks by Hebbian learning (Nagai et al., 2011; Kawai et al., 2012) and neural networks (Ogata et al., 2009; Arie et al., 2012; Nakajo et al., 2015). Through training, experimenters observe behavior changes in robots and in the internal states of the learning models, then consider the developmental processes of imitation. The Hebbian learning approach reveals changes in granularity on visual development, allowing the robot to recognize self–other correspondences (Nagai et al., 2011; Kawai et al., 2012). Our previous studies used recurrent neural networks to demonstrate how robots can translate from other to own actions (Ogata et al., 2009), imitative ability for the composition of behaviors (Arie et al., 2012), and recognition of positional relations between self and other (Nakajo et al., 2015).

For robots working in human living environments, imitation of demonstrator behaviors roughly comprises two processes: (1) observing the behavior and (2) transforming the observed behavior into an action. During observations, robots are expected to extract information about the imitated behavior. In the transformation process, robots must extract necessary information from the observations, and match them with their own actions. Robots cannot always observe behaviors from the same position, but are expected to recognize and reproduce behaviors regardless of the position from which they were observed. However, few previous studies have focused on positional relations between robots and demonstrators or considered correspondences between actions provided from various positions.

If robots are to observe demonstrated actions and transform them into the robots' own actions, robots must process raw images and extract from them information necessary for imitation. However, the huge dimensionality of raw data makes direct processing too difficult. Deep-learning techniques are looked to as a solution to this problem (LeCun et al., 2015), because deep learning can process raw data and allows machines to automatically extract necessary information about requested tasks. For instance, deep learning techniques have outperformed previous methods for image recognition (Krizhevsky et al., 2012). Over the past several years, deep learning has been applied to action learning by robots, and many studies have investigated imitative learning through deep learning (Liu et al., 2017; Sermanet et al., 2017; Stadie et al., 2017). Stadie et al. applied deep learning methods to transformation of demonstrator views into robot control features. Sermanet et al. and Liu et al. trained learning models to relate demonstrator views from various positions with the robot view. After training learning models to transform demonstrator views, reinforcement learning (Liu et al., 2017; Stadie et al., 2017) or supervised learning methods (Sermanet et al., 2017) are applied to allow robots to imitate behaviors. Although these learning methods are suited to allowing robots to acquire imitative skills regardless of positional relations, demonstrators cannot provide their views to robots in actual environments; robots must instead capture demonstrator behaviors via cameras, and relate observed behaviors to their own situation.

Various training methods have also been researched in the field of deep learning. One common method applied to robot action learning is end-to-end learning, in which the learning model receives images and robot motor commands, and directly plans the robot's actions. Another technique often applied to natural language translation is sequence to sequence learning (Sutskever et al., 2014), which allows translation of a multi-dimensional time series into another time series. Utilizing this characteristic, Yamada et al. (2016) allowed a robot to perform tasks based on language instructions. This characteristic can also be applied to imitative learning, because robots must translate observations of demonstrator actions into their own actions. We thus consider the application of sequence to sequence learning to imitative learning.

The main contribution of this paper is demonstration of how a robot can acquire the following two abilities: (1) automatic visual-feature extraction, and (2) transformation from human demonstration into robotic action when positional differences are present. This paper proposes an imitative learning model that simultaneously enables a robot to acquire positional relations between a demonstrator and the robot, and transforms observed actions into the robot's own actions. In the learning process, the robot observes demonstrator actions using a mounted camera, and no pre-training is provided. To achieve imitative abilities, we combined two deep neural network models. An autoencoder extracts visual features from raw camera images, and a dynamic neural network model called a multiple timescale recurrent neural network (MTRNN) (Yamashita and Tani, 2008) is trained to learn how to imitate tasks. An MTRNN learns positional relations between a demonstrator and a robot. To allow the robot to learn how to translate observed actions into its own actions, the MTRNN is trained based on a sequence to sequence approach (Sutskever et al., 2014). In experiments, we imposed object manipulation tasks on a robot and conducted predictive learning to train the proposed learning model. After training, we confirmed that the robot could translate observed actions into its own actions. By inspecting the internal states of the MTRNN, we show how the robot recognizes positional relations between the demonstrator and the robot during tasks. We also considered what information the robot extracts through observation and translates into actions.

## 2. Methods

### 2.1. Sequence to sequence learning of imitative interaction

**Figure 1:**
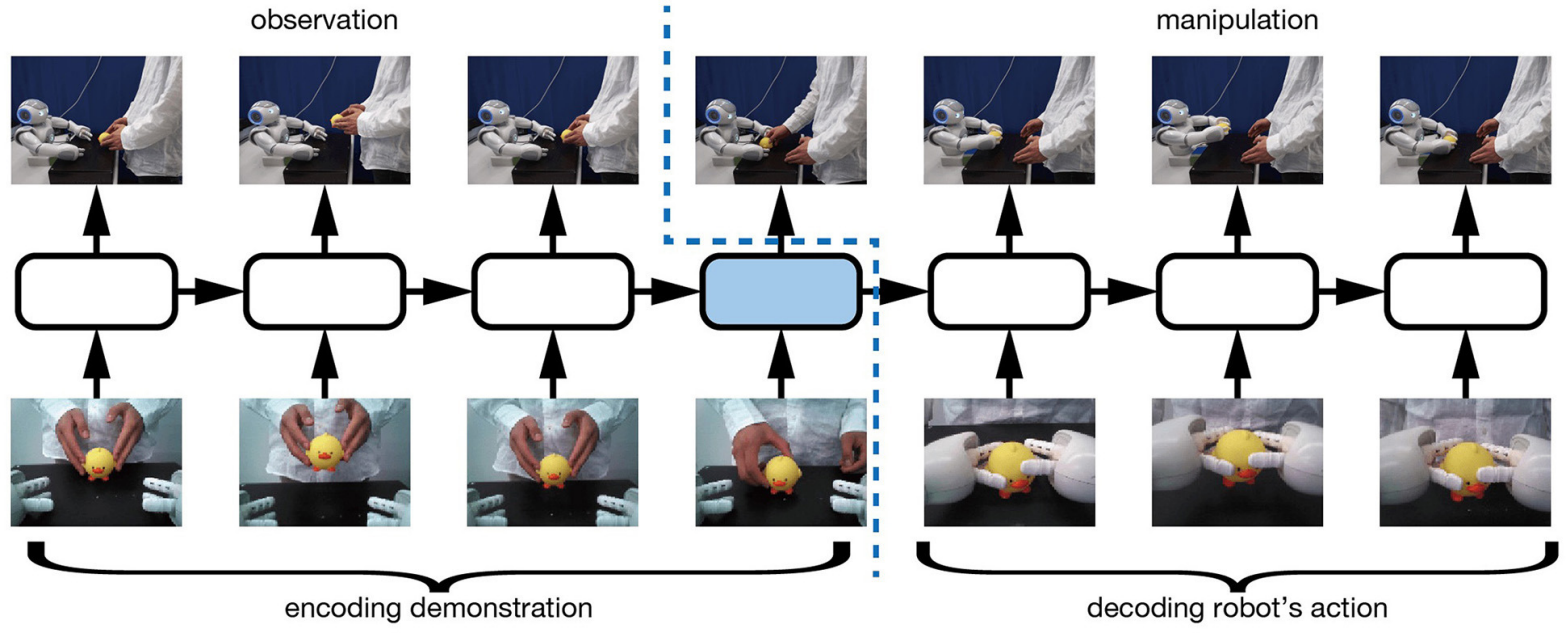
sequence to sequence learning scheme of the RNN. In the first half of the time sequence, the robot moves only its head and captures images of only the action being demonstrated. From the captured images, the RNN is expected to recognize and encode the demonstrator actions. In the second half of the time sequence, the RNN receives encoded internal states, plans robot actions, and issues robot motor commands.

We first describe the method by which robots use our proposed learning model to learn imitative interactions. We apply sequence to sequence learning (Sutskever et al., 2014) to map observed demonstrator actions to robot actions. sequence to sequence learning is a learning method for RNNs that is mainly used in the machine translation field. By inputting to RNNs series of sentences in the original and target languages, sequence to sequence learning allows forward propagation in the RNNs both to recognize the meaning of the original sentence and to generate a sentence in the target language by using the internal states acquired through encoding the original language. We use sequence to sequence learning to encode demonstrator actions and to generate robot actions. As Figure 1 shows, by concatenating demonstrator and robot actions and inputting the concatenated sequences to a RNN, the network is expected to learn how to map the demonstrator actions to robot actions.

### 2.2. Overview of proposed learning model

Robot imitation of demonstrator actions requires observation of demonstrator actions and transformation of observed actions into robot actions. The robot must process visual information to extract information related to demonstrator actions. Captured camera images have too many dimensions to process directly. The robot thus requires functions for automatically compressing and extracting visual information. To map extracted visual information from demonstrator actions to robot actions, visual features and robot motor information must be integrated into a single learning scheme. Doing so requires another learning model for integrating this information, separate from visual feature compression.

**Figure 2:**
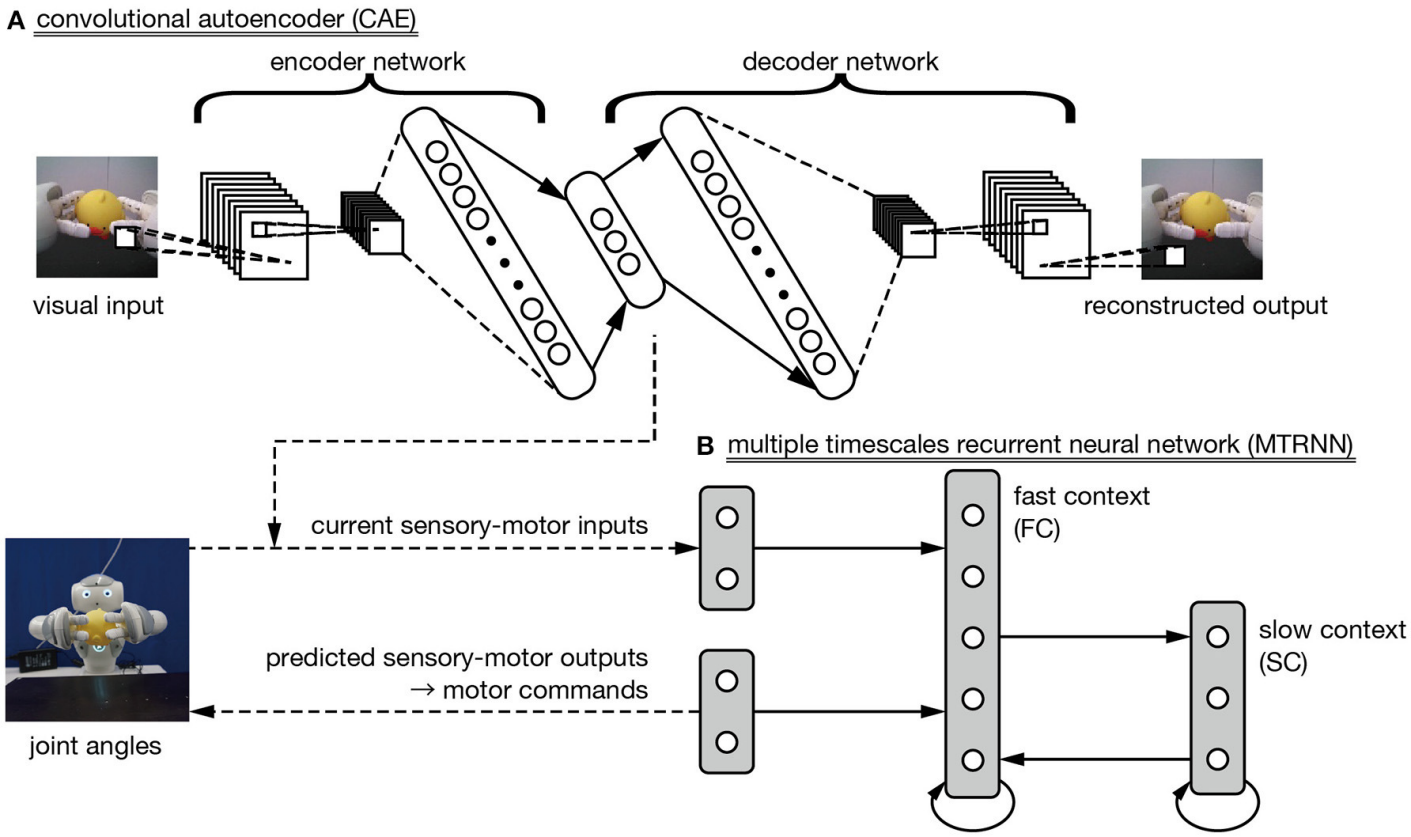
The proposed learning model. **(A)** A convolutional autoencoder (CAE) is trained to extract visual features in images from a robot-mounted camera. **(B)** A multiple timescale recurrent neural network is used to integrate CAE-extracted visual features and robot motor information.

Our proposed learning model satisfies these conditions by including two neural networks. The first is a deep neural network called a convolutional autoencoder (CAE), which is applied to extraction of visual features from camera images. The second is a multiple timescale recurrent neural network (MTRNN), which we use to integrate time series of extracted visual features with robot motor information. Figure 2 shows an overview of the proposed learning model. In the following subsections, we explain the CAE method for extracting visual features and the MTRNN method for integrating them with motor information.

### 2.3. Visual feature extraction via convolutional autoencoder

An autoencoder is a neural network with bottleneck layers, and comprises an encoder for dimensionally compressing input images and a decoder for restoring dimensionality in output images (Hinton and Salakhutdinov, 2006). Updating learnable parameters in the autoencoder to identically output an input image allows the network to acquire lower-dimensional features representing input images at the narrowest layer. By compressing input images, the robot can nondestructively extract visual features of camera images.

In this study, we applied a convolutional autoencoder (CAE), which is an autoencoder including convolution layers (Masci et al., 2011). Convolution is an arithmetic process inspired by the mammalian visual cortex, and is expected to extract visual features by focusing on spatial localities in the images. We combined a conventional CAE with fully connected layers. Camera images are taken as input, then the CAE is trained to minimize the mean squared error between input and reconstructed images. The mean squared error *E*_AE_ is processed as

(1) $$\begin{array}{l} E_{\text{AE}}=\frac{1}{N}\overset{N}{\underset{n}{\sum }}E_{\text{AE}}^{\left(n\right)}, \end{array}$$

(2) $$\begin{array}{l} E_{\text{AE}}^{\left(n\right)}=\frac{1}{HWC}||{\hat{\text{X}}}^{\left(n\right)}-{\text{X}}^{\left(n\right)}|{|}_{2}^{2}, \end{array}$$

where *N* is the number of mini-batches; ${\hat{\text{X}}}^{\left(n\right)}$ is the *n*th input image; **X**^(*n*)^ is the *n*th reconstructed image; and *H*, *W*, and *C* indicate the height, width, and channel, respectively, of the images. To avoid drastic changes in extracted visual features between continuous time steps, we furthermore applied the following slow penalty introduced in Finn et al. (2016):

(3) $$\begin{array}{l} g\left({\text{f}}_{t}\right)=\eta \cdot ||\left({\text{f}}_{t+2}-{\text{f}}_{t+1}\right)-\left({\text{f}}_{t+1}-{\text{f}}_{t}\right)|{|}_{2}^{2}\;\left(t\geq 1\right), \end{array}$$

where **f**_*t*_ indicates the visual features extracted from an image at time step *t*, and η is a hyper-parameter to control the strength of the penalty.

### 2.4. Sensory-motor integration by multiple timescale recurrent neural network

Generating imitative actions from observation of demonstrator actions requires a function that integrates visual features extracted by the CAE with robot motor information. In this work, we use a dynamic neural network model called a multiple timescales recurrent neural network (MTRNN) (Yamashita and Tani, 2008). An MTRNN has different time constants in its hierarchically context layers. The layer connected to the input–output layers [“fast context” (FC) in Figure 2B] is a group of neurons with a smaller time constant, and so responds more quickly to current external inputs. Another layer connected only to neurons in the context layers [“slow context” (SC) in Figure 2B] has a larger time constant, and so responds more slowly. Yamashita and Tani (2008) demonstrated that stacking layers with different timescales allows the robot to acquire action primitives in the FC layer, and described the order of sequential combinations of primitives in the SC layer.

In MTRNN forward propagation, the internal state of the *i*th FC, SC, and output neural unit at time step *t*, (*u*_*t, i*_), for the *s*th sequence is calculated as

(4) $$u_{t,i}^{\left(s\right)}=\{\begin{array}{l} \left(1-\frac{1}{\tau_{i}}\right)u_{t-1,i}^{\left(s\right)}+\frac{1}{\tau_{i}}\left(\sum_{j\in I_{\text{I}}}w_{ij}x_{t,j}^{\left(s\right)}+\sum_{j\in I_{\text{FC}}\cup I_{\text{SC}}}w_{ij}c_{t-1,j}^{\left(s\right)}+b_{i}\right)\;\left(t\geq 1,i\in I_{\text{FC}}\right),\\ \left(1-\frac{1}{\tau_{i}}\right)u_{t-1,i}^{\left(s\right)}+\frac{1}{\tau_{i}}\left(\sum_{j\in I_{\text{FC}}\cup I_{\text{SC}}}w_{ij}c_{t-1,j}^{\left(s\right)}+b_{i}\right)\;\left(t\geq 1,i\in I_{\text{SC}}\right),\\ \sum_{j\in I_{O}}w_{ij}c_{t,j}^{\left(s\right)}+b_{i}\;\left(t\geq 1,i\in I_{\text{O}}\right), \end{array}$$

where *I*_FC_, *I*_SC_, and *I*_O_ are index sets of the respective neural units, τ_*i*_ is the time constant of the *i*th neuron, *w*_*ij*_ is the connective weight from the *j*th to the *i*th neural units, $x_{t,j}^{\left(s\right)}$ is the external input of the *j*th neural unit at time step *t* of the *s*th sequential data, $c_{t,j}^{\left(s\right)}$ is the activation value of the *j*th context neuron at time step *t* of the *s*th sequence, and *b*_*i*_ is the bias of the *i*th neural unit. We use tanh as the activation function for the context neural unit $c_{t,i}^{\left(s\right)}$ and output unit $y_{t,i}^{\left(s\right)}$.

We trained the MTRNN by minimizing the mean squared error with the gradient descent method. The mean squared error *E*_RNN_ is described as

(5) $$\begin{array}{l} E_{\text{RNN}}=\frac{1}{S}\overset{S}{\underset{s}{\sum }}\frac{1}{T^{\left(s\right)}}\overset{T}{\underset{t}{\sum }}E_{\text{RNN},t}^{\left(s\right)}, \end{array}$$

(6) $$\begin{array}{l} E_{\text{RNN},t}^{\left(s\right)}=\frac{1}{Y}||{\hat{\text{y}}}_{t}^{\left(s\right)}-{\text{y}}_{t}^{\left(s\right)}|{|}_{2}^{2}, \end{array}$$

where *S* is the number of sequential data, *T*^(*s*)^ is the number of time steps of the *s*th sequential data item, *Y* is the number of neural units in the output layer, ${\hat{\text{y}}}_{t}^{\left(s\right)}$ is the target sensory-motor values at time step *t* of the *s*th sequence, and ${\text{y}}_{t}^{\left(s\right)}$ is the predicted sensory-motor values at time step *t* of the *s*th sequence. The learnable parameters of the MTRNN are composed of connected weights **w**, biases **b**, and initial internal states in context layers ${\text{u}}_{0}^{\left(s\right)}$. The gradients of these learnable parameters follow a conventional back propagation through time method (Rumelhart et al., 1986).

## 3. Experiment

### 3.1. Task design

**Figure 3:**
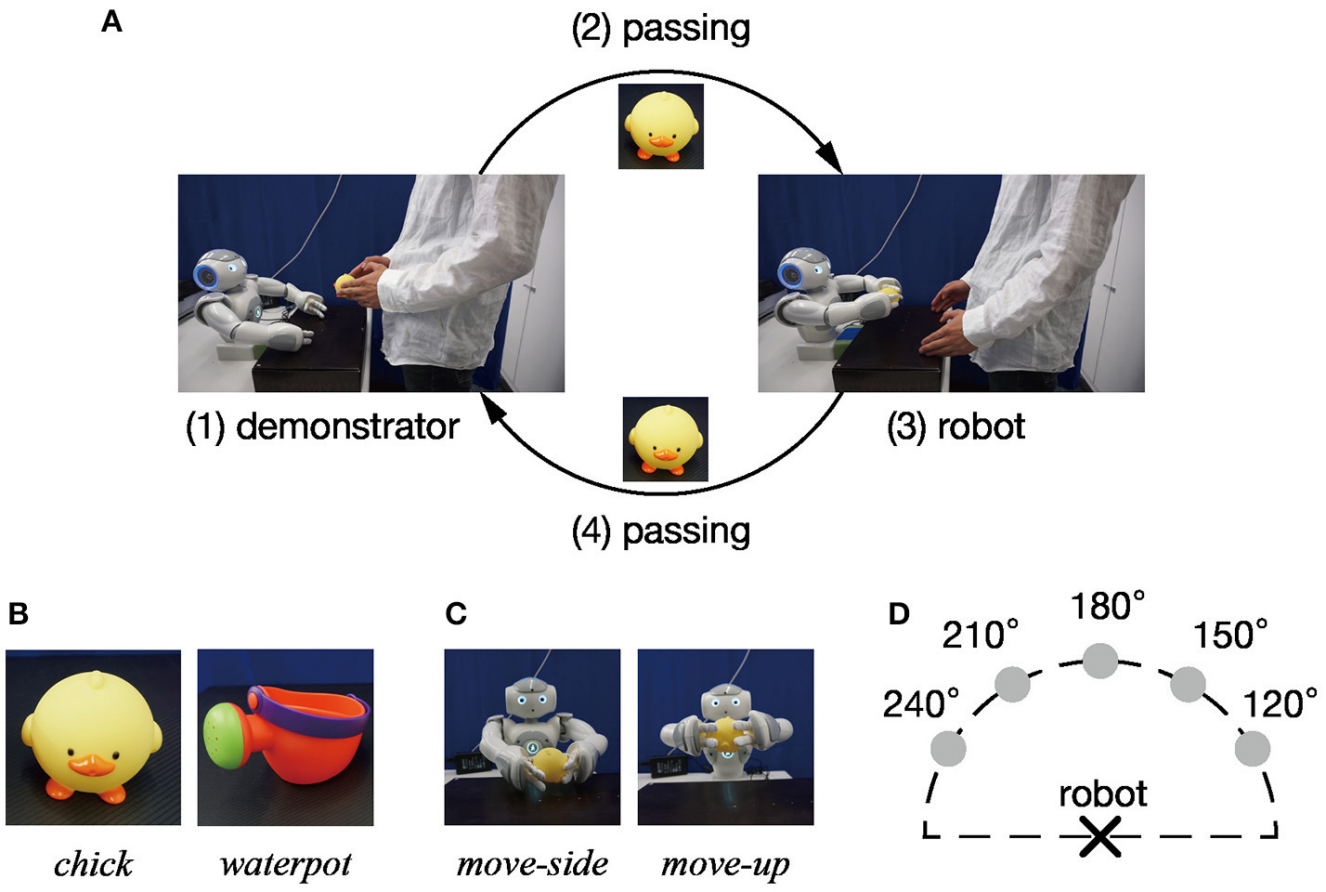
Task design: **(A)** A single cycle of the imitative interaction task given to the robot comprises four components: (1) action presentation by the demonstrator, (2) passing the manipulated object to the robot, (3) generating an imitative action by the robot, and (4) receiving the object by the demonstrator. **(B)** Objects manipulated during imitative interaction. **(C)** Imitative robot actions. **(D)** Positional relation between robot and demonstrator. The position of actions that the robot observed in front of the demonstrator is defined as 180°, and five positions (120, 150, 180, 210, and 240°) are labeled counterclockwise.

This section describes an experimental task given to a humanoid robot (NAO; Aldebaran Robotics). The task in this experiment is imitative interaction for object manipulation as shown in Figure 3A. Imitative interaction cycles comprised four processes: (i) the demonstrator shows the object manipulation action to the robot, then (ii) passes the manipulated object to the robot. Next, (iii) the robot mimics the observed manipulation, and (iv) the demonstrator receives the object from the robot. Furthermore, actions, manipulated objects, and positional relationships between the robot and the demonstrator were varied between cycles. Manipulated objects were two toys (a *chick* and a *watering can*), shown in Figure 3B. Objects were manipulated in two ways (*move-side* and *move-up*) as shown in Figure 3C. The positional relationship between the robot and the demonstrator varied according to where the demonstrator presented the action. We define 180° as the position when the robot presents a motion in front of itself. Accordingly, 120, 150, 180, 210, and 240° counterclockwise in the positive direction are used as the positional relationship between the demonstrator and the robot. Figure 3D shows a schematic diagram of positional relations between the demonstrator and the robot. Under these conditions, combinations that can be taken in a single cycle come in 20 patterns, from two objects, two movements, and five positional relations.

### 3.2. Training data

This subsection describes the method for creating sequential training data. In this experiment, the training data consisted of time series of the robot joint angles and 120 × 160 RGB images captured by a front-facing camera mounted in its mouth. The CAE extracts visual features from captured images. Controlled joints had four degrees of freedom (DoF) (ShoulderPitch, ShoulderRoll, ElbowYaw, and ElbowRoll) at each arm and two DoF (HeadPitch and HeadYaw) at the neck.

To prepare the training data, the robot was controlled and actual joint angles and images were recorded. A control method for both arms was predesigned and the arms tracked the planned trajectories with noise. Gaussian noise was added into the planned trajectories to augment the training data, with the noise variance set as 0.0001. Neck joint angles were operated by proportional–integral–derivative control, so manipulated object centroids were centered in camera images during interaction. While recording training data, joint angles and camera images were sampled every 400 ms. Because recorded joint angle and camera image information had different value ranges, the information was normalized before input to the neural networks: joint angles were scaled to [−1.0, 1.0] according to angle limits, and image pixel values were normalized from [0, 255] to [−1.0, 1.0].

This experiment separately recorded the processes of imitative interaction tasks such as demonstrator and robot actions and object passing. After recording, processes were combined and an imitative interaction cycle was generated. There were 160 time steps for demonstrator and robot actions and 60 for passing objects between the demonstrator and the robot, for a total of 440 time steps. Each sequence of 20 combinations was generated five times, for a total of 100 instances of recorded data.

### 3.3. Training of CAE and MTRNN

The robot was trained with imitative interaction tasks through predictive learning of recorded time series including joint angles and camera images.

#### 3.3.1. Visual feature learning via CAE

We first trained the CAE with camera images to extract visual features for input to the MTRNN with robot joint angles. Input 120 × 160 RGB images have 57,600 dimensions. These input images were trained to minimize errors between the original inputs and reconstructed images, and to extract 10 visual features from the middle CAE layer. Table 1 presents the detailed CAE structure used in this learning experiment. For CAE training, we conducted mini-batch training with an Adam optimizer (Kingma and Ba, 2015), setting Adam hyperparameters as α = 0.01, β_1_ = 0.9, and β_2_ = 0.99, mini-batch sizes of 200, and slow penalty strength as η = 1.0 × 10^−5^. Learnable CAE parameters were updated 7,500 times.

**Table 1:** The structure of the CAE.

**The *l*th layer**	**Input**	**Output**	**Processing**	**Kernel size**	**Stride**	**Padding**
1	(120, 160, 3)	(60, 80, 16)	Conv	(4, 4)	(2, 2)	(4, 4)
2	(60, 80, 16)	(30, 40, 32)	Conv	(4, 4)	(2, 2)	(4, 4)
3	(30, 40, 32)	(10, 10, 64)	Conv	(6, 8)	(3, 4)	(6, 8)
4	(10, 10, 64)	(2, 2, 128)	Conv	(10, 10)	(5, 5)	(10, 10)
5	512	250	Linear	−	−	−
6	250	10	Linear	−	−	−
7	10	250	Linear	−	−	−
8	250	512	Linear	−	−	−
9	(2, 2, 128)	(10, 10, 64)	Deconv	(10, 10)	(5, 5)	(10, 10)
10	(10, 10, 64)	(30, 40, 32)	Deconv	(6, 8)	(3, 4)	(6, 8)
11	(30, 40, 32)	(60, 80, 16)	Deconv	(4, 4)	(2, 2)	(4, 4)
12	(60, 80, 16)	(120, 160, 3)	Deconv	(4, 4)	(2, 2)	(4, 4)

*In the “Processing” column, conv, deconv, and linear respectively indicate convolutional encoding, deconvolutional decoding, and fully-connected transformation. The input dimensions for convolutional and deconvolutional layers are shown as (height, width, channel), and fully-connected layers are shown as d*.

#### 3.3.2. Sensory-motor integration learning via MTRNN

After extracting visual features by the trained CAE, time series of sensory-motor information were generated by concatenating robot joint angles and extracted visual features. To allow the robot to carry out imitative interactions, training sequences for input to the MTRNN were created by connecting several combinations of imitative tasks. In this case, training sequences were sequences of four randomly selected imitative tasks, with overlapping allowed. An interval of 5–30 time steps was inserted between the connected time series. The robot retained the same pose during this interval. Under these conditions, 100 sequences were generated as MTRNN training data.

While there were 20 combinations of imitative tasks, we trained the MTRNN with 10 combinations to evaluate generalizability to unlearned combinations. Table 2 shows the 10 combinations used for MTRNN training to predict the next state of joint angles and visual features. There were 10 joint angles and 10 extracted visual features, for a total of 20 dimensions input to the MTRNN. We set the number of neural units in the FC and SC layers as 180 and 20 and time constant values as 2.0 and 64.0, respectively. For training, we used the Adam optimizer with hyperparameters α = 0.01, β_1_ = 0.9, and β_2_ = 0.99. Learnable parameters were updated with these settings 10,000 times.

**Table 2:** MTRNN training sequences.

	**120°**	**150°**	**180°**	**210°**	**240°**
*move-side*	C	W	C	W	C
*move-up*	W	C	W	C	W

*Rows show actions, and columns show positional relationships. In each cell, characters C and W indicate the manipulated object (chick or watering can). The time sequence indicated in each cell is used for MTRNN training*.

## 4. Training results

### 4.1. Reconstructed images by CAE

**Figure 4:**
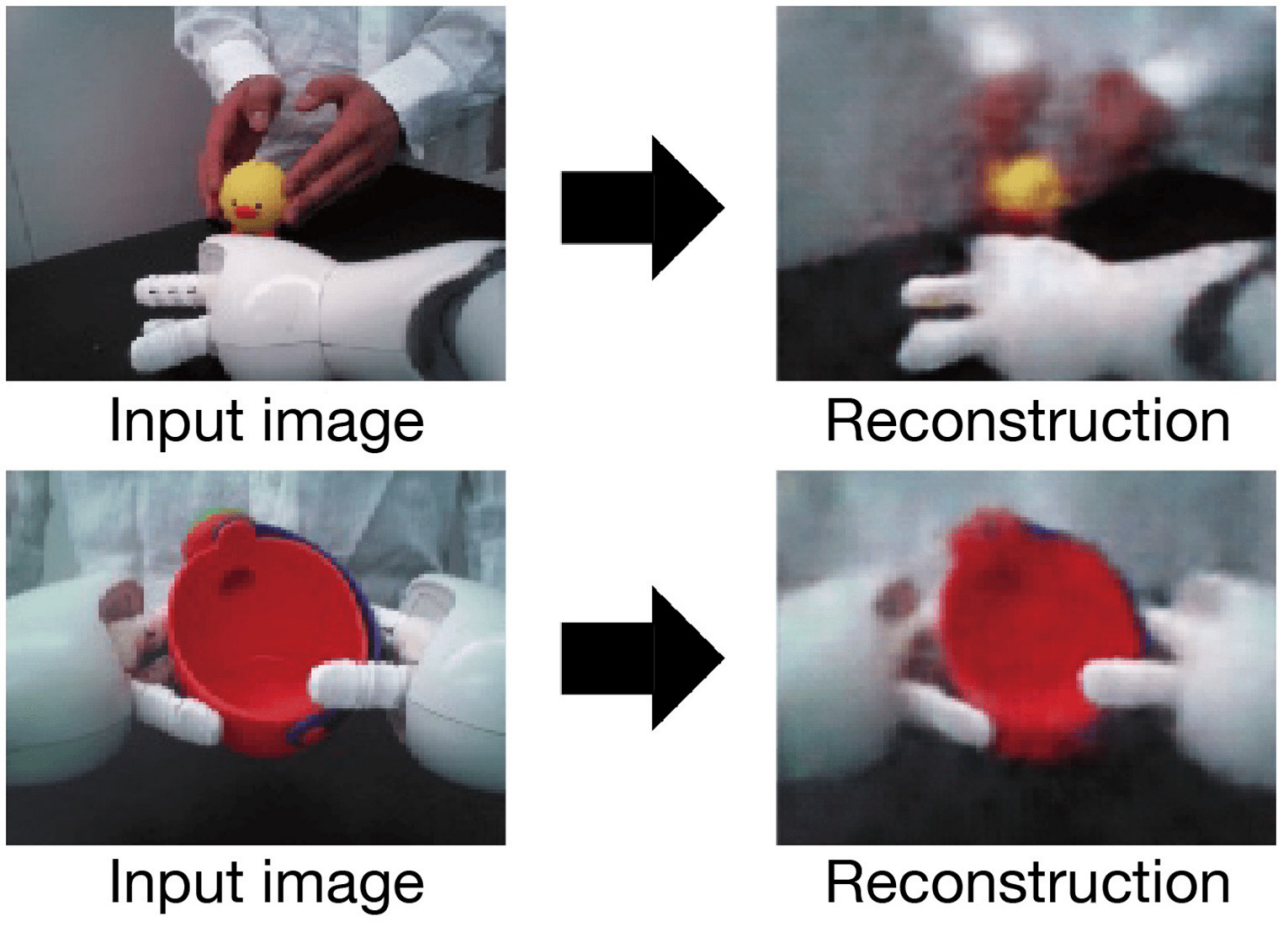
Image reconstruction by trained CAE. The upper figure shows an image of a [move-side, *chick*] demonstration, and the lower figure shows an image of [*move-up, watering can*] generated by the robot.

**Figure 5:**
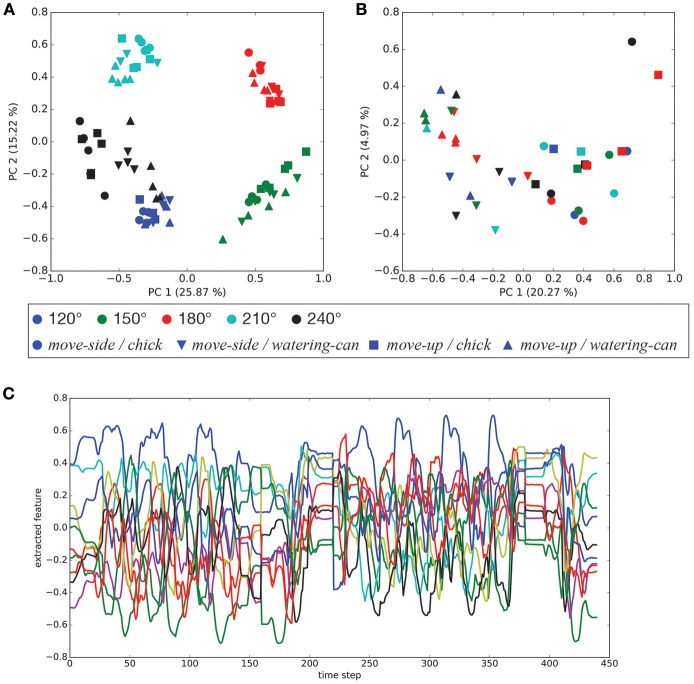
Visual features extracted by the CAE: **(A)** principal components of the visual features at the beginning of demonstrations (PC1–PC2), **(B)** principal components of the visual features at the beginning of robotic actions (PC1–PC2), and **(C)** an example of a time series of the visual features for [*move – side, watering – can*, 150°].

After CAE training, the mean squared error between trained images and their reconstructed output was at most 0.0141. The worst mean squared error between untrained and reconstructed images was 0.0150. Figure 4 shows a selection of reconstructed and untrained images. The reconstructed image in Figure 4 suggests that the trained CAE could regenerate original input images. We applied principal component analysis to visual features extracted by the CAE at the beginnings of the demonstrations and robotic actions. As shown in Figure 5A, the positional relationships between the demonstrator and the robot were separated in the visual features at the beginning of the demonstrations. Figure 5B shows that the manipulated objects were separated in the visual features at the beginning of the robotic actions. The CAE could extract the visual features from images, thus we used time series of the extracted visual features for training of the MTRNN. An example of a time series of the extracted visual features is shown in Figure 5C.

### 4.2. Robot action generation

**Figure 6:**
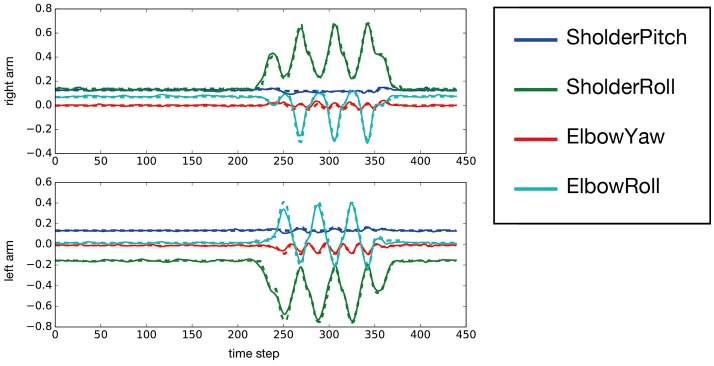
The predicted output of an untrained [*move – side, chick*] sequence observed from the 150° position. This figure shows only the prediction for both arms. The horizontal axis indicates time steps, and the vertical axis represents predicted output of the joint angles. The solid and dotted lines show output by the MTRNN and target sequences, respectively.

After MTRNN training, we evaluated the mean squared error between trained target sequences and predicted output, which was 0.00140 at worst. We input new sequences generated with the combination including untrained series, and evaluated the mean squared error. In that case, the evaluated value was 0.00164 at worst. Figure 6 shows the MTRNN-predicted output against the untrained input [*move*−*side, chick*] as observed from position 150°. By using predicted output of the MTRNN against untrained input, the robot could imitate demonstrator actions.

### 4.3. Internal states in MTRNN

**Figure 7:**
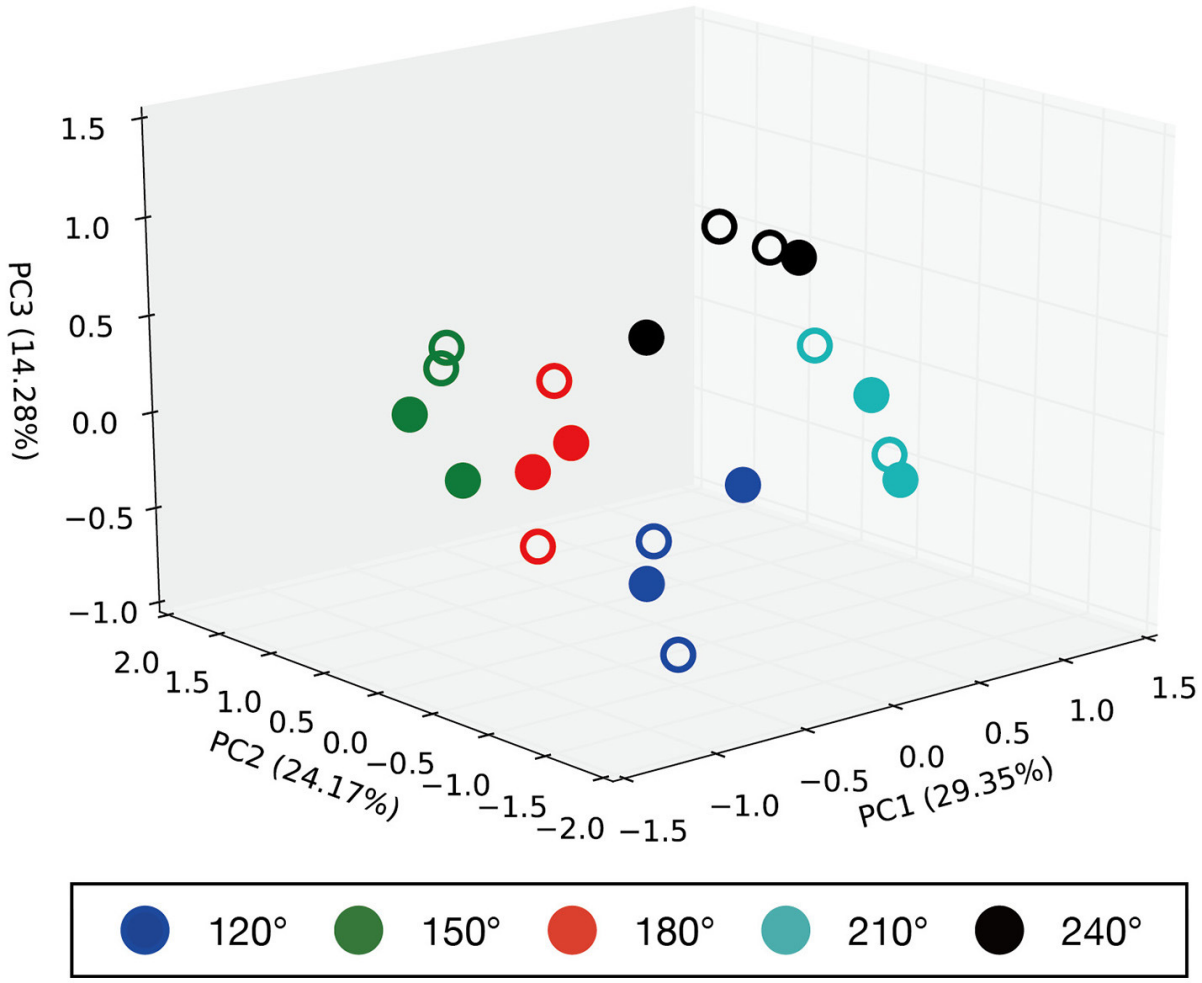
Results of PCA of the internal states in the FC layer when demonstrator actions are finished. PC1, PC2, and PC3 are plotted in the 3D space. Numbers in parentheses indicate contribution ratios of each principle component. Filled points are trained imitative patterns, and others are unlearned patterns. The positional relationships are separated in the 3D space.

**Figure 8:**
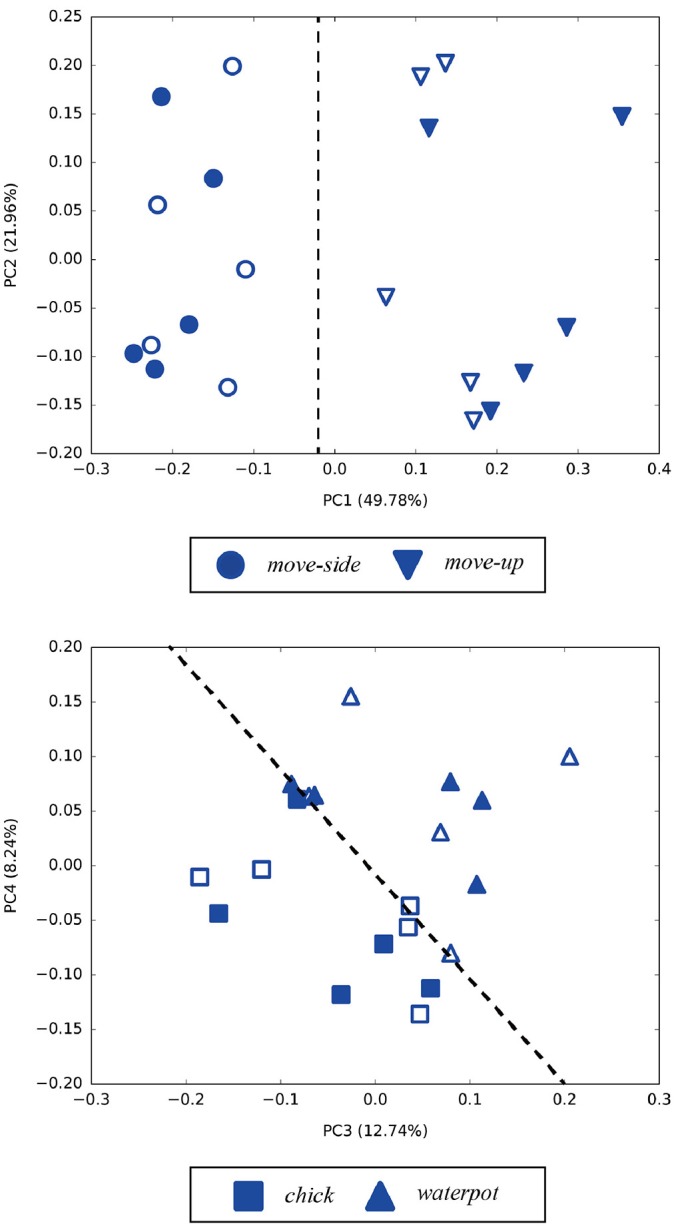
Results of PCA of internal states in the SC layer when demonstrator actions are finished. Numbers in parentheses indicate contribution ratios of each principle component. Filled points are trained imitative patterns, and others are unlearned patterns. In the upper figure (PC1–PC2), differences of actions are separated in the PC1 direction. In the lower figure (PC3–PC4), differences of manipulated objects are classified by the dashed line.

Principal component analysis was performed on the internal MTRNN state to grasp the internal structure the MTRNN acquired through predictive learning of robot sensory-motor information. We conducted PCA on internal states in the FC and SC layers at the time when the demonstrator ended the actions. Figure 7 shows the difference in the positional relationship between the demonstrator and the robot in the FC layer, and Figure 8 shows the difference between imitative actions and manipulated objects. As shown in Figure 7, the FC layer in the MTRNN separated positional relationships between the robot and the demonstrator when demonstrator actions were complete. At the same time, differences in imitative actions are clustered in the plane described by PC1 and PC2 of the internal states in the SC layer (see the upper graph in Figure 8). In contrast, in the plane described by PC3 and PC4 the differences between manipulated objects are separated by the dashed line in the lower graph in Figure 8.

**Figure 9:**
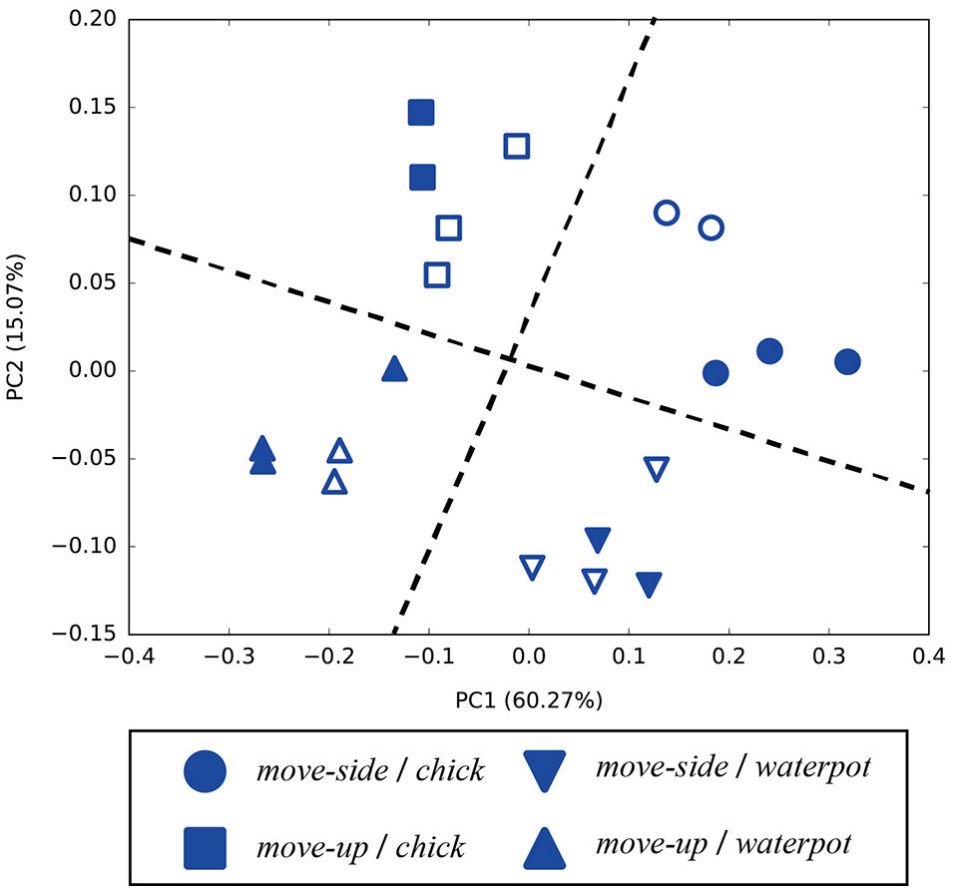
Internal states in the SC layer at the beginning of robot actions (PC1–PC2). Filled points indicate trained imitative patterns, and outlined marks are unlearned. Combinations of imitative actions and manipulated objects can be clustered by the two dotted lines.

We next extracted internal states in the SC layer at the time when the robot starts its action, and plotted the PCA results in Figure 9. As that figure shows, combinations of imitative actions and manipulated objects were clustered in the SC layer. The actions were distinguished at the beginning of robot imitation, so the robot could map observed actions to corresponding imitative actions in advance. Similarly, the robot could acquire an ability to carry out imitative actions while retaining information about manipulated objects in the internal MTRNN states. Furthermore, unlearned patterns indicated in Figure 9 were recognized, so the MTRNN could acquire the ability to generalize via combinations of actions and manipulated objects.

**Figure 10:**
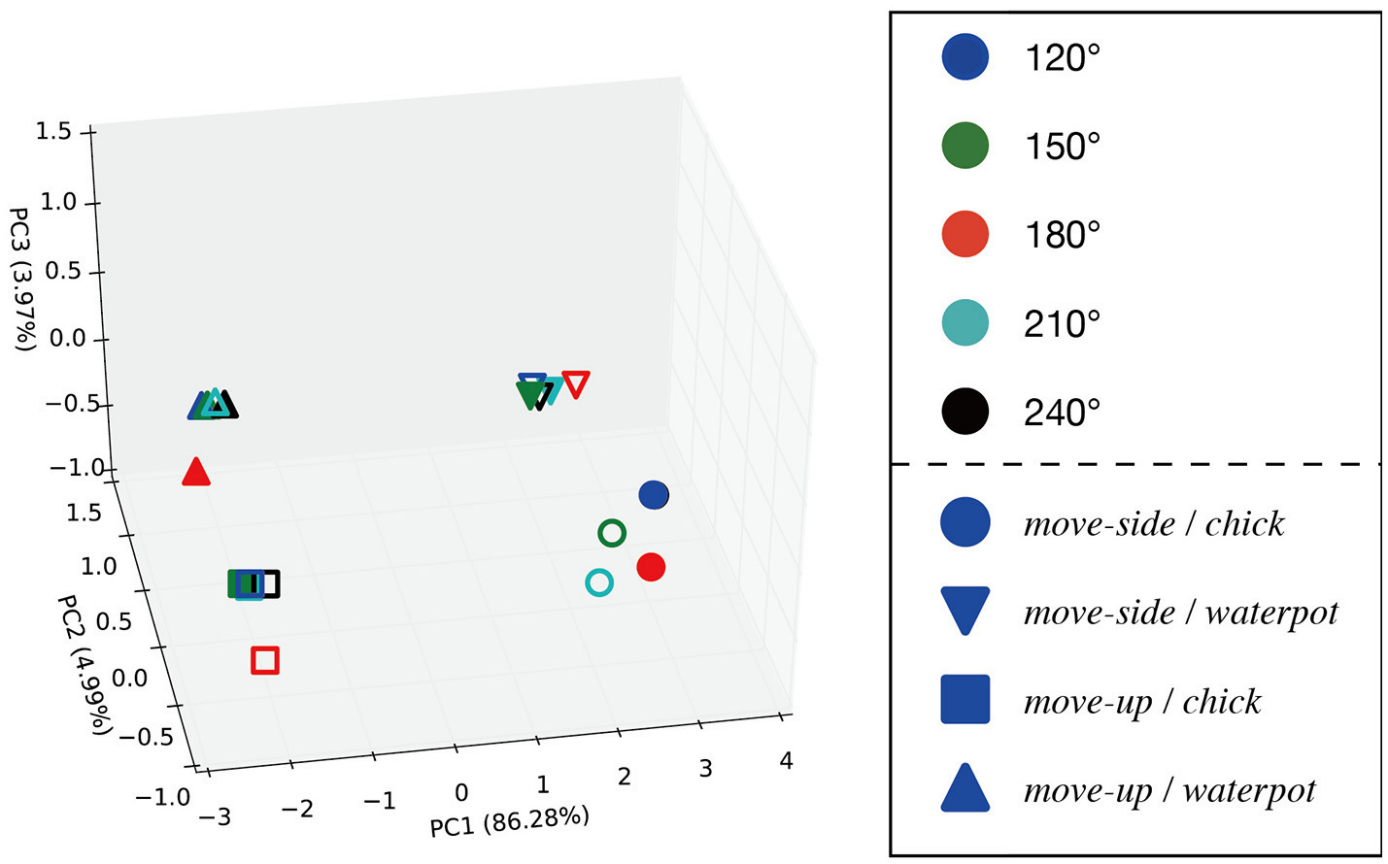
Internal states in the FC layer while conducting robot actions (PC1–PC2–PC3). Filled points indicate trained imitative patterns, and outlined marks are unlearned patterns. Actions and objects are distinguished between in this 3D space, but positional differences between the demonstrator are ignored.

**Figure 11:**
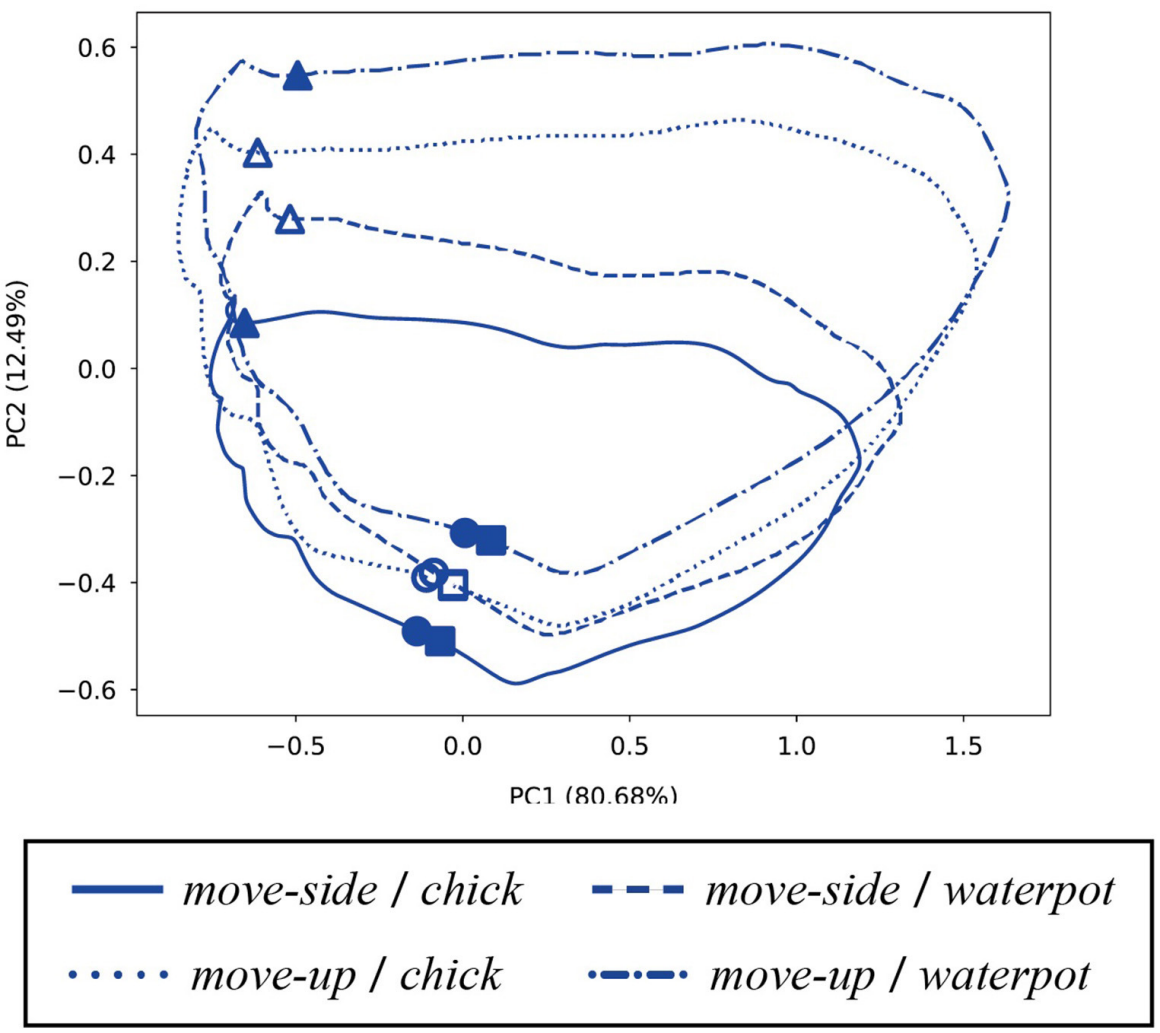
Transition of neural activities in the SC layer during imitative interaction (PC1–PC2). The positional relationship between the demonstrator and the robot is fixed as 120°, and combinations of actions and manipulated objects are separately plotted. Symbols ○, △, and □ respectively indicate the beginning of demonstrator actions, the beginning of robot actions, and the end of passing objects to the demonstrator. Filled marks indicate trained patterns, and others indicate unlearned patterns. All transitions start from ○ points, pass through △, and finally return to similar □ points near the beginning of demonstrator actions (○).

One time step during the robot action was chosen and the internal states were analyzed at that time. Since robot motions comprised 160 steps, we chose the middle (80th) time step and visualized the internal states by PCA. Figure 10 shows internal states of the FC layer at that time, and confirms that the robot distinguished between different combinations of actions and manipulations while performing imitative actions. In contrast, principle components in the FC layer do not show positional relations between the demonstrator and the robot. Therefore, the robot could transform observations into actions regardless of the positional relation. Finally, to confirm how internal MTRNN states transit during imitative interaction, we plotted the time development of neural units in the SC layer during interaction in a plane. Figure 11 shows transitions of neural activities in the SC layer during imitative interactions. The positional relationship between the demonstrator and the robot is fixed as 120°, and combinations of actions and manipulated objects are separately shown. The figure shows that the internal states for all patterns start from the beginning of demonstrator actions (○), transit to robot actions (△), and finally reach the same point where manipulated objects are passed from the robot to the demonstrator (□). Since the internal states always reach the same point, the robot could continue to recognize the actions, manipulated objects, and positional relations after a single imitative interaction. Other positional relations also acquired results similar to those in Figure 11.

## 5. Discussion

We proposed a possible imitative model that allows a robot to acquire the ability to recognize positional relations between the demonstrator, and to transform observed actions into robot actions. The imitative model had two neural networks: (1) a CAE that was trained to extract visual features from captured raw images, and (2) an MTRNN that integrated and predicted sensory-motor information. Through training of image reconstruction by the CAE, the robot could extract visual features from raw images captured by its camera. By sensory-motor integration through predictive learning with the MTRNN, the robot could recognize information that relates imitative interactions, such as positional relations between the demonstrator and the robot. In the rest of this section, we compare earlier studies with our current work, and clarify the distinction between them.

From the viewpoint of acquiring positional relations between the demonstrator and robot, our proposed model allows the robot to recognize positional relations via predictive learning of sensory-motor sequences. By including differences in positions between the demonstrator and the robot, the proposed learning model might be forced to optimize these differences during predictive learning. Thanks to the hierarchical structure of the MTRNN and the sequence to sequence learning methods, the robot might come to process positional differences in the FC layer (shown in Figure 7), and possess information required for robot actions, such as kinds of actions and manipulated objects in the SC layer (see Figures 8, 9). In this work, the sequence to sequence learning method was tried for encoding the demonstrator's actions into the plan of robotic actions. Thus, the information necessary for the robotic actions may be encoded in the SC layer, and the information necessary for the current prediction may appear in the FC layer. In the current experiment, the robotic actions do not require any positional relationships between the demonstrator and the robot. Therefore, positional relationships may remain in the FC layer. Furthermore, from Figure 10, conducting sequence to sequence learning that translates demonstrator actions into robot actions might allow the robot to properly transform observed actions into the same actions. In previous works, positional relations between demonstrator and robot were represented by coordinate transformations described as mathematical formulations (Billard et al., 2004; Lopes et al., 2010). Our proposed model requires no designed transformation to acquire positional relations between the demonstrator and robot. In this experiment, the robotic head moved through imitative interaction, and its joint angles differed for each positional relationship during the demonstration phase. These difference in the robotic head depended on the positional relationships between the demonstrator and the robot. Thus, the proposed learning model might require optimizing for these differences during predictive learning. Through predictive learning of sensory-motor sequences, including positional differences between the demonstrator and robot, the robot could automatically recognize differences and transform demonstrator actions into robot actions. Our previous work (Nakajo et al., 2015) allowed robots to acquire information about actions and positional relations by labeling this information and providing constraints that make activities of neural units representing the same information close. In contrast, the current work eliminates labeling of actions and positional relations by conducting sequence to sequence learning.

From the perspective of action translations, sequence to sequence learning methods might contribute to learning how to translate demonstrator actions into robot actions. As Figures 7, 8 show, the robot recognized positional relations, actions, and manipulated objects in the demonstration phase. From Figure 10, after a demonstration, the robot could perform observed actions regardless of positional relation. Thanks to the characteristics of sequence to sequence learning, which can translate one multidimensional sequence into another sequence, the robot acquired the ability to choose information necessary for conducting actions. In addition, we conducted a validation trial in which the demonstrations from untrained positional relationships (135, 165, 195, and 225°) were given to the MTRNN. The demonstrations observed from all untrained positions could be translated into the proper robotic actions by the MTRNN. On the other hand, although the MTRNN could map the untrained positional relationships into the points between the trained positional relationships, sometimes mapping failed and these relationships appeared at different points in the PCA space of Figure 7. These failures might come from visual features extracted by the CAE. In the current experiment, differences in the positional relationships were present in the visual images and the joint angles of the robotic head. However, the CAE did not learn to extract visual features from the untrained positional relationships. Thus, it may be difficult to extract these visual features with the CAE, which could affect predictions by the MTRNN. Previous studies applied separate modules to transform positional differences (Ogata et al., 2009; Liu et al., 2017; Sermanet et al., 2017). Positional relations that the robot recognized were thus limited by the number of experts, although the robot could imitate observed actions from various positions. In this paper, every positional relationship is acquired within the internal structure of a single RNN, so the robot can process various positional relations. Sermanet et al. (2017) and Liu et al. (2017) used deep neural networks that associated demonstrator views with robot views. These methods were very powerful, because no previous knowledge was required to associate the views. However, third-person views were synchronized with robot views where needed to translate actions. In this paper, the robot required its own views, so a robot-mounted camera was necessary in an actual environment. Furthermore, from the viewpoint of transforming actions, previous works used separate modules to extract invariances that were included in views, and additional training was required to learn robot actions. Our proposed model allowed the robot to simultaneously learn recognition of positional relations and action transformation, so no pre-training was needed to integrate sensory-motor information.

When we train the CAE to extract visual features from the robot's vision, we discretely input visual frames. However, in sensory-motor integration for achieving sequential tasks, visual feature learning in which the learning model sequentially predicts images may be required. In the experiment described in this paper, robot actions were determined at the end of the demonstration, and only passing of objects occurs between the end of demonstrator actions and the beginning of robot actions. Thus, both internal representations in the SC layer might be similar. However, discrimination of manipulated objects was not acquired at the end of demonstrator actions, as shown in Figure 8. Discrimination of manipulated objects was instead achieved at the beginning of robot actions, as shown in Figure 9. This difference in representations might come from prediction error arising from visual information. For the CAE, the difficulty of reconstructing any object comes from the size of object regions. Specifically, reconstructing smaller objects is more difficult than larger objects. In this paper, the regions of manipulated objects during demonstration are smaller than those during robot actions. It thus seems more difficult for the CAE to reconstruct manipulated objects in the demonstration phase. This difficulty of reconstruction might affect sensory-motor integration, as seen in the internal representations in the SC layer. Video prediction in which the learning model is trained to sequentially predict images would contribute to overcoming this problem. Thanks to sequential prediction, the learning model applies histories of past predictions to the current prediction. Moreover, we separately trained the CAE and the MTRNN. Therefore, through training of sensory-motor integration with the MTRNN, no feedback was sent to visual processing by the CAE. However, to allow the robot to more properly process sensory-motor sequences, the prediction error should affect all processing in the learning model. A previous work by Hwang and Tani (2017) prepared a neural network that processes visual sequences, and another that controls the robot. By combining two neural networks through another subnetwork, they realized end-to-end training of sensory-motor integration. Our learning model has a structure similar to the model proposed by Hwang and Tani (2017), so combining two neural networks through another subnetwork might also be applicable to the proposed method.

We conducted sequence to sequence learning to allow the robot to transform each demonstrator action into robot actions. However, by giving the learning model pairs of demonstrator and robot actions that differ from the demonstrator's, sequence to sequence learning can realize translation of demonstrator actions into robot actions differing from the demonstrator's. Furthermore, we gave only one-to-one pairs of demonstrator and robot actions as training data during sequence to sequence learning. The robot can thus only imitate demonstrated actions in a single way, and cannot acquire imitative ability that performs demonstrated actions with equivalent goals but conducted by differing means, such as using both hands vs. using only one hand. Such an imitative ability is important for robots, but has not yet been realized by current methods using sequence to sequence learning. To realize this imitative ability, in future studies we should enrich training data to allow the robot to imitate demonstrated actions by various means. In the training data, the demonstrator and robot conduct equivalent actions by various means. Through training pairs of demonstrator and robot actions, the robot might come to imitate demonstrated actions in various ways. As has been found in the field of neural machine translation (Cho et al., 2014; Johnson et al., 2016), RNNs with an encoder–decoder architecture trained by sequence to sequence learning methods can acquire both syntactic and semantic structures. Thus, by applying sequence to sequence learning to action learning by robots, RNNs might allow robots to capture the underlying structures of demonstrated actions.

In this paper, imitative learning using a sequence to sequence learning method required an RNN to deal with long sequences. Therefore, RNNs other than MTRNN could be used to learn sensory-motor sequences. For example, we tried a continuous-time recurrent neural network (CTRNN) for the current experiment. Although the CTRNN generated the trained imitative patterns after predictive learning, it sometimes failed to generate untrained imitative patterns. As another example, it is well known that the long short-term memory technique (LSTM) can process long sequences because of its gating mechanisms. Thus, replacing MTRNN with LSTM will yield similar results. Although an RNN other than MTRNN could have been used, we adopted MTRNN because of its simpler representation of the internal state.

Moreover, future studies from the viewpoint of imitative learning should discuss the existence of mirror neurons (Rizzolatti et al., 1996), which by themselves show common ignition states in the imitation ability of primates with the perception of other acts and movement. This mirror neuron system has also been discussed from the viewpoint of cognitive development robotics, because human beings lead the development of behavioral understandings in others (Nagai et al., 2011; Arie et al., 2012; Kawai et al., 2012). In a previous study (Nakajo et al., 2015), we realized robot acquisition of common neuronal transitions in the robot's own and other behaviors by constraint to neurons representing labeled information, but the internal states of all neurons were separated according to their own actions in this work. Therefore, as a future method for realizing neuron activity simulating mirror neurons, it is conceivable to consider an imitation experiment using a group of neurons with slow response speeds in the context layer of the RNN.

## Author contributions

RN, SM, HA, and TO conceived, designed the research, and wrote the paper. RN performed the experiment and analyzed the data.

### Conflict of interest statement

The authors declare that the research was conducted in the absence of any commercial or financial relationships that could be construed as a potential conflict of interest.
